# Metabolic conversion of CI-1040 turns a cellular MEK-inhibitor into an antibacterial compound

**DOI:** 10.1038/s41598-018-27445-7

**Published:** 2018-06-14

**Authors:** Christin Bruchhagen, Marcel Jarick, Carolin Mewis, Tobias Hertlein, Silke Niemann, Knut Ohlsen, Georg Peters, Oliver Planz, Stephan Ludwig, Christina Ehrhardt

**Affiliations:** 1 Institute of Virology Muenster (IVM), Westfaelische Wilhelms-University Muenster, Von-Esmarch-Str. 56, D-48149 Muenster, Germany; 2 Institute for Molecular Infection Biology (IMIB), University of Wuerzburg, Josef-Schneider-Str. 2/D15, D-97080 Wuerzburg, Germany; 3 Institute of Medical Microbiology, University Hospital of Muenster, Domagkstr. 10, D-48149 Muenster, Germany; 4 Interfaculty Institute for Cell Biology, Department of Immunology, University of Tuebingen, Auf der Morgenstelle 15, D-72076 Tuebingen, Germany

## Abstract

Influenza virus (IV) infections cause severe respiratory illnesses that can be complicated by bacterial super-infections. Previously, we identified the cellular Raf-MEK-ERK cascade as a promising antiviral target. Inhibitors of MEK, such as CI-1040, showed potent antiviral activity. However, it remained unclear if this inhibitor and its active form, ATR-002, might sensitize host cells to either IV or secondary bacterial infections. To address these questions, we studied the anti-pathogen activity of ATR-002 in comparison to CI-1040, particularly, its impact on *Staphylococcus aureus* (*S*. *aureus*), which is a major cause of IV super-infections. We analysed IV and *S*. *aureus* titres *in vitro* during super-infection in the presence and absence of the drugs and characterized the direct impact of ATR-002 on bacterial growth and phenotypic changes. Importantly, neither CI-1040 nor ATR-002 treatment led to increased bacterial titres during super-infection, indicating that the drug does not sensitize cells for bacterial infection. In contrast, we rather observed reduced bacterial titres in presence of ATR-002. Surprisingly, ATR-002 also led to reduced bacterial growth in suspension cultures, reduced stress- and antibiotic tolerance without resistance induction. Our data identified for the first time that a particular MEK-inhibitor metabolite exhibits direct antibacterial activity, which is likely due to interference with the bacterial PknB kinase/Stp phosphatase signalling system.

## Introduction

Influenza viruses cause infections of the respiratory tract resulting in severe diseases, especially in high-risk patients. Fast illness progression and high mortality rates are often associated with secondary bacterial infections induced by common colonizers of the nasopharynx, such as *S*. *aureus* or *Streptococcus pneumoniae* (*S*. *pneumoniae*)^1–5^. In contrast to infections with streptococci that occur at late phases following viral clearance, *S*. *aureus* is mostly detected during concomitant IV infections. Furthermore, *S*. *aureus* gives rise to a continuously growing challenge in the clinics due to resistance development, such as Methicillin-resistant *S*. *aureus* (MRSA) strains^6–8^. Similarly, resistant IV variants evolve and vaccine evasion occurs regularly. Thus, there is an urgent need for new therapeutics against both pathogens^9–13^.

Novel antiviral strategies to combat influenza are based on the fact that IV, as intracellular pathogens, strongly depend on the cellular signalling machinery^14^. Thus, cellular virus-supportive functions are promising candidates for alternative approaches thereby reducing the likelihood to provoke viral resistance. In contrast, *S*. *aureus* division has been mostly thought of as host-cell independent. By directing novel antibacterial treatments towards inhibition of bacterial virulence factors expressed during infection, these compounds may also exhibit a lower potential to induce resistance. Additionally, there is accumulating evidence that *S*. *aureus* also uses cellular signalling for its own benefits during infection^15^, but such bacterial-supportive cellular factors have yet to be characterized.

The cellular Raf-MEK-ERK pathway is involved in the nuclear export of newly synthesized viral ribonucleoproteins (vRNPs) during IV replication. Prior studies have shown that compounds inhibiting this pathway exhibit significant anti-influenza activity *in vitro* and *in vivo*^16,17^. The inhibition of this pathway by use of specific MEK-inhibitors like U0126 and CI-1040 resulted in a reduction of viral titres without induction of resistances^11,16–19^. Especially CI-1040 was shown to be a potent compound for anti-influenza intervention with a prolonged treatment window when compared to standard of care^18^. *In vivo* CI-1040 is metabolized into its acidic form ATR-002 (originally termed PD0184264), which is the major initial metabolite after oral administration and appears to be the biological active entity^20,21^. Yet, the anti-pathogen activity of ATR-002 has not previously been tested.

One concern of using cellular signalling inhibitors is sensitization of a flu-infected host to subsequent super-infections. In fact, the activation of the Raf-MEK-ERK pathway and downstream signalling cascades has been demonstrated upon bacterial infections^22,23^ and Raf-MEK-ERK-mediated signalling appears to be important in immune responses during singular IV and bacterial super-infection with *S*. *aureus* and *S*. *pneumoniae*^23,24^.

It was assumed for a long time that serine/threonine (Ser/Thr) kinases, like MEK and ERK, are exclusively expressed by eukaryotes, but recently eukaryotic-like Ser/Thr kinases have been identified in the majority of bacterial families^25^. These prokaryotic kinases are involved in various cellular functions, like metabolic processes, cell wall metabolism, environmental responses and pathogenicity^26–28^. Notably, *S*. *aureus* expresses the Ser/Thr kinase PknB, that shares high homology with cellular MAPK^29^. Besides metabolic processes, PknB is involved in regulation of bacterial antibiotic susceptibility and other pathogenicity determining processes including stress response and growth behaviour^30–34^.

One aim of the present study was to elucidate whether MEK-inhibitors, such as CI-1040 and its metabolite ATR-002, would enhance replication of bacteria during IV/*S*. *aureus* super-infection. Furthermore, we aimed to explore the anti-pathogen activity of ATR-002, especially the direct impact on bacterial growth.

## Results

### Treatment with CI-1040 or ATR-002 does not sensitize cells for secondary bacterial infections

**Figure 1:**
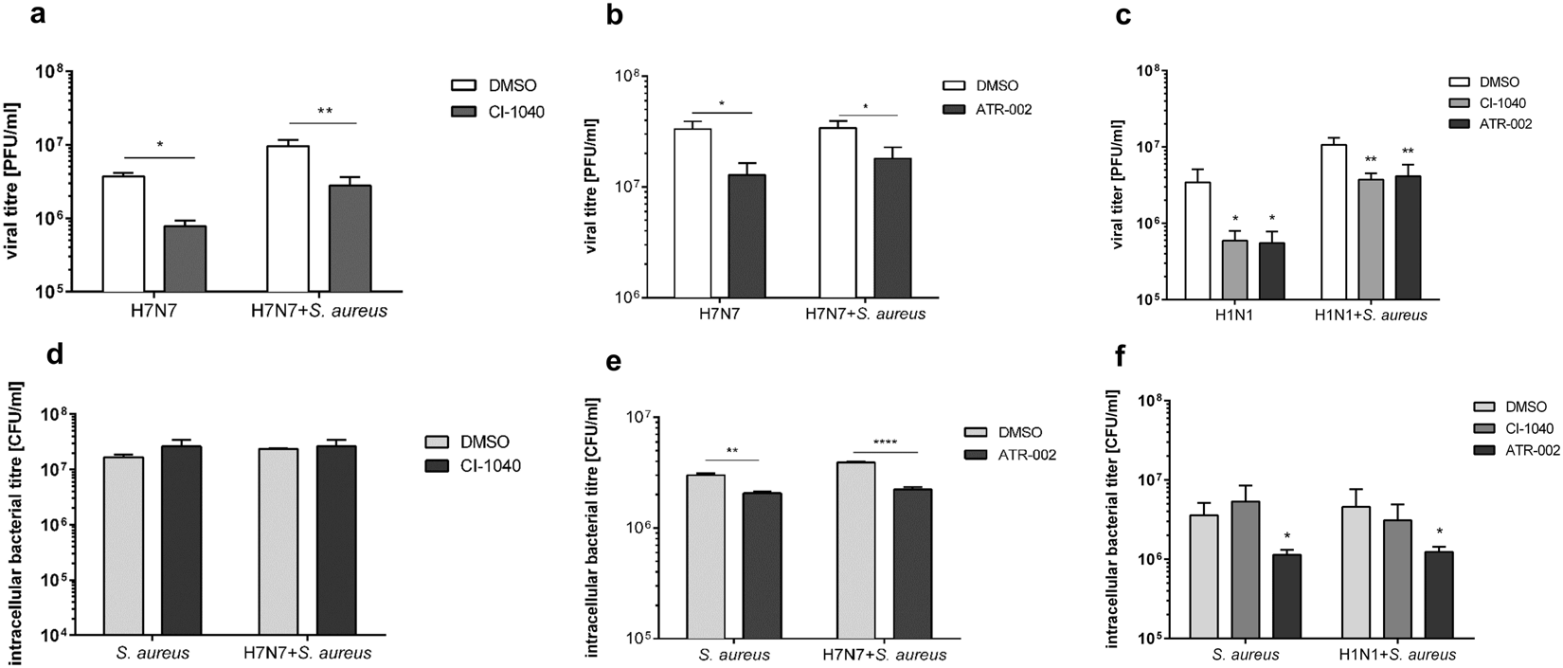
ATR-002, but not CI-1040, decreases intracellular bacterial titres. A549 cells were pre-treated (**a**,**b**,**d**,**e**) with 10 µM CI-1040, ATR-002 or solvent (DMSO) for 60 min and then infected with IV (H7N7) at a MOI of 0.001 at 37 °C. Alternatively, cells were left untreated (**c**,**f**) and infected with IV (H1N1) at a MOI of 0.01 at 37 °C. After 30 min the virus dilution was removed, cells were rinsed with PBS and supplemented with invasion medium with or without *S*. *aureus* 6850 (6850) (MOI 0.1) in the presence of 10 µM CI-1040, ATR-002 or solvent control. 3 h post bacterial infection cells were treated with lysostaphin (2 µg/mL) for 20 min to remove extracellular bacteria. Cells were then washed and supplemented with infection medium containing the inhibitor or solvent. After a total incubation period of 24 h (post viral infection) viral (**a**–**c**) and intracellular bacterial titres (**d**–**f**) were analysed. Results represent means + SD of three individual experiments. Statistical significance was evaluated by one-way ANOVA followed by Tukey’s multiple comparisons test (*p < 0.05; **p < 0.01; ***p < 0.001; ****p < 0.0001).

IV infection results in enhanced expression of antiviral cytokines, most importantly type I IFNs, that activate critical downstream antiviral responses and may also potentiate subsequent bacterial infections^2,35–37^. Since the Raf-MEK-ERK pathway is involved in expression of some of these cytokines we wondered whether treatment with CI-1040 or ATR-002 would sensitize cells for a secondary *S*. *aureus* infection. Cell cultures of immortalized human alveolar basal epithelial cells (A549) were infected with IV and *S*. *aureus* in the presence or absence of the inhibitors. Inhibitors were used at 10 µM, which is considered to be a concentration still specific for MEK inhibition in cell culture^38^. Microscopic examination revealed that super-infection with both pathogens resulted in a highly increased cytopathic effect (CPE) compared to singular infections (Figure S1, upper panel). The CPE was completely abolished in presence of ATR-002 (Figure S1, lower panel) indicating reduced viral replication. Similar results were obtained upon MEK inhibition where both CI-1040 and ATR-002 reduced IV titres, with a less pronounced effect of ATR-002 (Fig. 1a,b). Due to variations between experiments, the effects of CI-1040 and ATR-002 on viral replication may not be strong, nevertheless, the effects seen were reproducible. Importantly, treatment with CI-1040 did not sensitize cells for a secondary infection with *S*. *aureus* as no changes in intracellular bacterial load could be detected (Fig. 1d). Surprisingly, administration of ATR-002 even resulted in reduced intracellular bacterial titres (Fig. 1e). Comparable results were obtained when CI-1040 or ATR-002 were administered at later times during on-going infection (Fig. 1c,f). To rule out that the reduction in viral and intracellular bacterial replication was a result of a cytotoxic effect of ATR-002 on A549 cells, cell viability in presence of increasing concentrations was monitored for 24 and 48 hours. Additionally, a LDH-Assay was performed to determine membrane rupture due to inhibitor treatment (Figure S2). Furthermore, we excluded a potential impact of ATR-002 on bacterial infectivity by performance of bacterial internalization assays in presence or absence of the inhibitor (Figure S3).

### *S*. *aureus* growth in suspension cultures is strongly inhibited by ATR-002, but only slightly affected by other MEK-inhibitors

**Figure 2:**
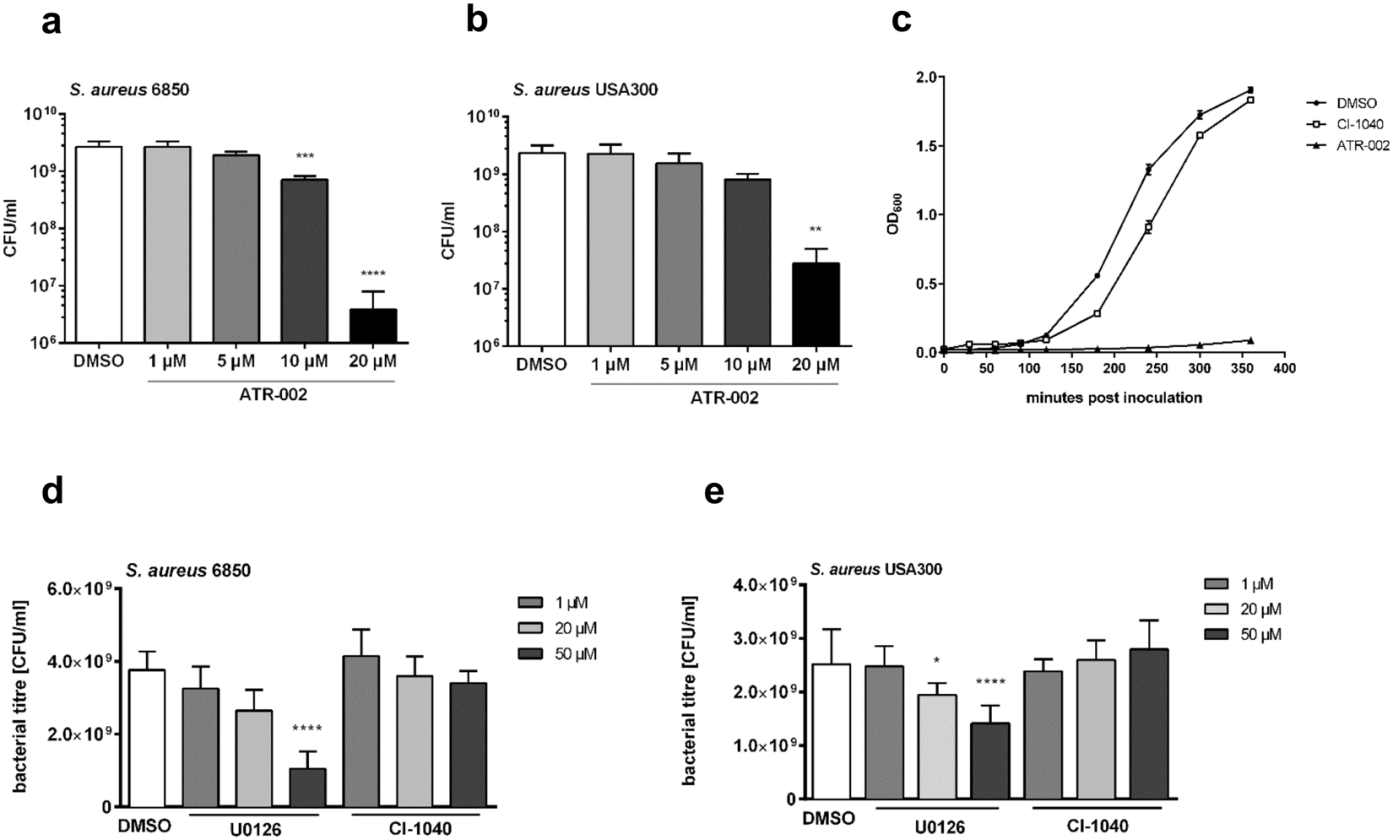
*S*. *aureus* growth is strongly impaired exclusively in presence of ATR-002. Over-day cultures of *S*. *aureus* 6850 or the MRSA strain USA300 were set to 20 CFU/ml and treated with different MEK-inhibitors (as indicated) over-night at 37 °C and 5% CO_2_. The OD_600_ was measured and remaining cultures were washed once with PBS. Bacterial titres were determined by serial dilutions on BHI agar plates (**a**,**b**,**d**,**e**). Growth curves were generated by dilution of 25 µl of untreated over-night cultures in 8 ml of fresh medium and were incubated over a time course of 6 h in presence of 50 µM CI-1040, ATR-002 or DMSO. The OD_600_ was measured at the indicated time points (**c**). Results represent means + SD (**a**,**b**,**d**,**e**); +/− SD (**c**) of three independent experiments. Statistical significance was analysed by one-way ANOVA followed by Dunnett’s multiple comparisons test (*p < 0.05; **p < 0.01; ***p < 0.001; ****p < 0.0001).

Upon administration of ATR-002 intracellular *S*. *aureus* titres were slightly reduced. While *S*. *aureus* does not rely on internalization into cells, bacteria express kinases homologous to eukaryotic MAPK. Consequently, we examined whether the reduced bacterial titres were an indirect result of the inhibited Raf-MEK-ERK pathway or due to direct interaction of ATR-002 with the bacteria. To assess this, we analysed the impact of increasing concentrations of ATR-002 and other MEK-inhibitors (U0126 and CI-1040) on suspension cultures of the Methicillin-sensitive *S*. *aureus* strain 6850 (MSSA) or the MRSA strain USA300 and calculated viable bacteria. Surprisingly, the presence of ATR-002 provoked a strong reduction of bacterial growth of the MSSA and to a slightly lower extent of MRSA strain (Fig. 2a,b). This growth retention by ATR-002 was already detectable shortly after inoculation of bacterial cultures and by far exceeded the effects of the parental compound CI-1040 (Fig. 2c). During over-night treatment with even higher concentrations of CI-1040 no comparably strong growth inhibition was observed. Although a significant reduction of *S*. *aureus* growth could be detected using 50 µM of U0126, this growth retention was considerably low compared to growth inhibition caused by ATR-002 (Fig. 2d,e). To confirm a medium-independent effect of ATR-002 on bacteria, we repeated these experiments in different media (Figure S4).

### ATR-002 exhibits bacteriostatic properties without induction of resistance *in vitro*

**Figure 3:**
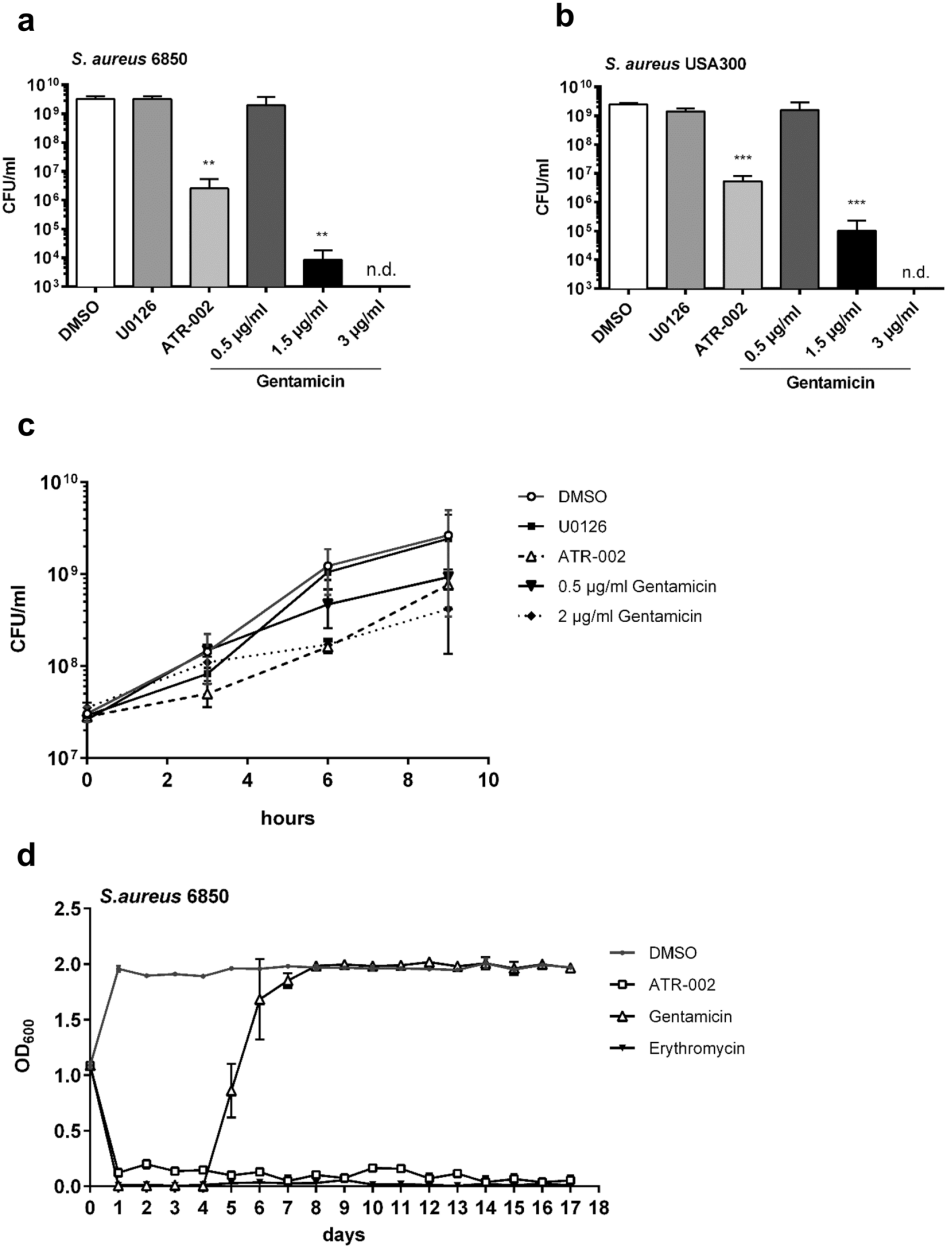
ATR-002 exhibits a low bacteriostatic effect without resistance induction. Over-day cultures of *S*. *aureus* 6850 or the MRSA strain USA300 were set to 20 CFU/ml and treated as indicated over-night at 37 °C and 5% CO_2_. The OD_600_ was measured, cultures were washed once with PBS and viable bacteria were counted by serial dilutions on BHI agar plates (**a**,**b**). To classify the antibacterial action, a time-of-addition assay was performed (**c**). Therefore, sub-cultures of an over-night culture of *S*. *aureus* 6850 were prepared and were treated for 9 h as indicated. At 0, 3, 6 and 9 h the OD_600_ was measured and titres were calculated. Additionally, long-term treatment with either ATR-002, Gentamicin or Erythromycin was performed to test for resistance development (**d**). Cultures were grown for 24 h in the presence or absence of the substances, the OD_600_ was measured and then cultures were set to 100 CFU/ml and grown again for 24 h. This procedure was repeated for 17 days. Additional results of testing for cross-resistance development are summarized in Table S1. Data represent means + SD of three independent experiments. Statistical significance was analysed by one-way ANOVA followed by Dunnett’s multiple comparisons test (*p < 0.05; **p < 0.01; ***p < 0.001; ****p < 0.0001).

To further classify the antibacterial action of ATR-002, growth behaviour of *S*. *aureus* was monitored in comparison to a known bactericidal agent, gentamicin. Treatment of MSSA and MRSA with 20 µM of ATR-002 led to a stronger reduction in bacterial titres than 0.5 µg/ml of gentamicin, but was less pronounced than treatment with 1.5 µg/ml of gentamicin (Fig. 3a,b).

Additionally, a time-of-addition assay was performed to investigate the action of ATR-002 on bacterial multiplication over time. Therefore, U0126, ATR-002 or gentamicin were added to *S*. *aureus* cultures. Bacterial amounts were analysed over a time course of 9 h starting at the time of compound addition. Interestingly, in the presence of ATR-002 inhibition of bacterial growth was even higher than caused by 2 µg/ml of gentamicin at early times of incubation (Fig. 3c). To shed some first light on the mode of action the bacterial cultures were diluted after 9 h in BHI medium and were further grown without substance addition. All cultures reached the turbidity of the untreated samples indicating a bacteriostatic rather than a bacteriocidal effect of ATR-002.

The frequent emergence of antibiotic resistant strains represents a major problem in the clinics. To test whether ATR-002 provokes resistance development, cultures were constantly treated for 17 days in presence of ATR-002, gentamicin or erythromycin. Resistance development to gentamicin occurred during the first week of treatment. In contrast, treatment with ATR-002 did not induce resistance similar to treatment with the macrolide erythromycin (Fig. 3d). Additionally, constant treatment with ATR-002 did not induce cross-resistances as shown by unchanged or even reduced MIC concentrations calculated for a variety of antibiotics after ATR-002 treatment (Table S1).

### ATR-002 treatment results in reduced antibiotic and stress resistance of *S*. *aureus*

**Figure 4:**
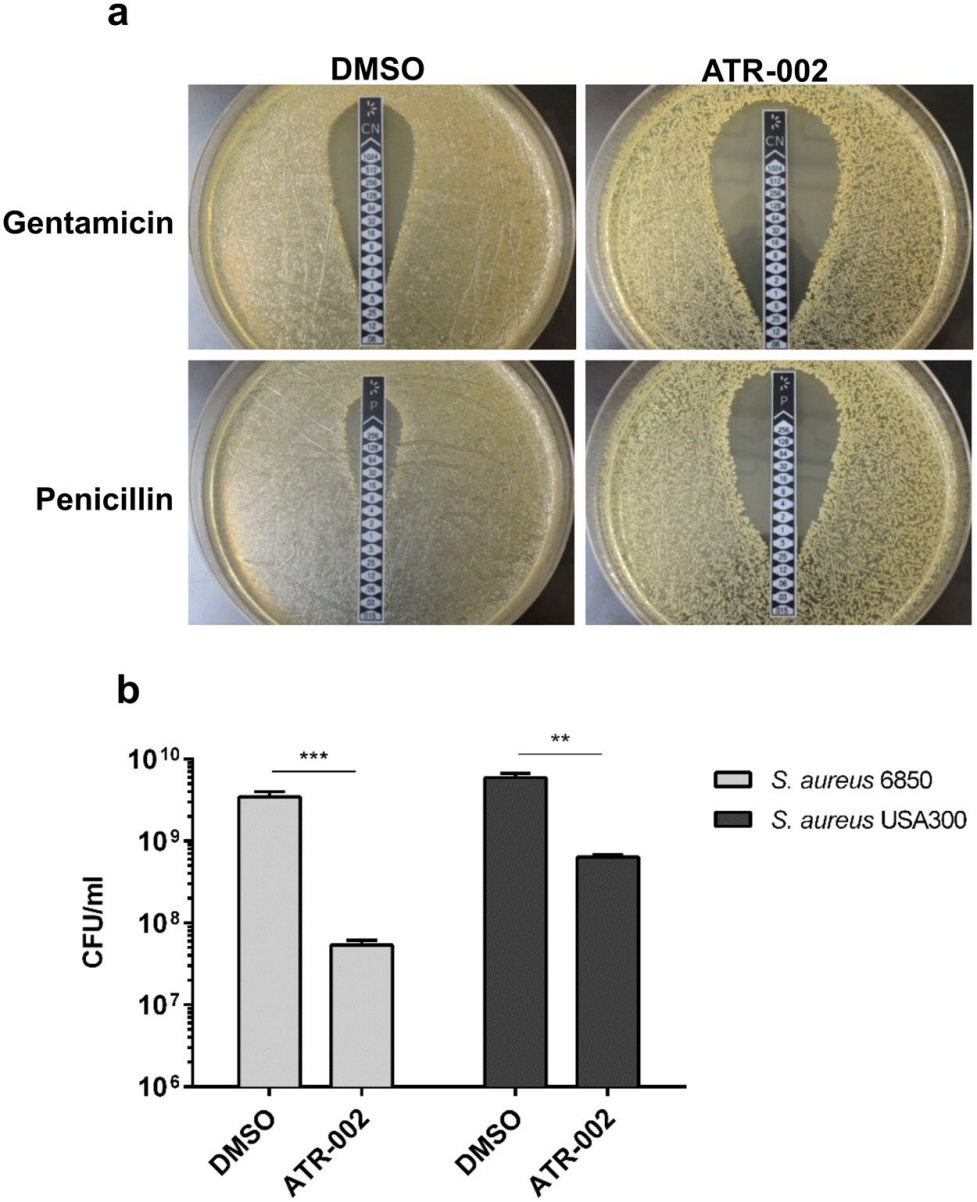
Treatment with ATR-002 results in increased antibiotic susceptibility and reduced stress resistance of *S*. *aureus*. Cultures of *S*. *aureus* 6850 (20 CFU/ml), originated from over-day cultures, were prepared in BHI medium and treated as indicated over-night at 37 °C. Subsequently, bacterial cultures were washed with PBS and resuspended in 1 ml PBS. MICs were determined using 100 µl of over-night cultures and a M.I.C.Evaluator strip on agar plates. After 24 h at 37 °C plates were analysed and the concentration preventing growth was termed as MIC (**a**). A summary of three independent experiments is shown in Table S2. Solvent or inhibitor treated over-night cultures were further cultivated after dilution in BHI medium at 42 °C to induce heat stress for 6 h. Then, bacterial counts were analysed (**b**). Data represent three individual experiments. Statistical significance was analysed by one-way ANOVA followed by Dunnett’s multiple comparisons test (**p < 0.01; ***p < 0.001).

Since ATR-002 is a cellular kinase inhibitor, bacterial Ser/Thr kinases, like PknB of *S*. *aureus*, would be a likely target candidate. *S*. *aureus* lacking PknB shows reduced growth, which correlates with increased antibiotic sensitivity and reduced stress tolerance^32,39^. Thus, we addressed the impact of ATR-002 on antibiotic susceptibility and heat tolerance of *S*. *aureus*. Therefore, we determined the MIC against different antibiotics after over-night treatment. Surprisingly, treatment with ATR-002 increased the susceptibility of both MSSA (*S*. *aureus* 6850) and MRSA (USA300) strains against various antibiotics, in particular penicillin susceptibility by up to tenfold. Here, representative data are shown for MSSA (Fig. 4a, Tables S2 and S3). Next, the ability of *S*. *aureus* to grow under stress conditions was evaluated after treatment. Therefore, the differentially treated cultures were set to an OD of 1, diluted in BHI and further incubated at 42 °C to induce heat stress. Compared to previously solvent-treated cultures, ATR-002-treated bacteria were more sensitive towards heat stress. Consequently, MSSA and MRSA strains showed a delayed growth (Fig. 4b). Heat stress response of these strains was further evaluated after treatment with ATR-002 in different growth media (Figure S5).

### The antibacterial action of ATR-002 is mediated by interaction with the PknB/Stp signalling system of *S*. *aureus*

**Figure 5:**
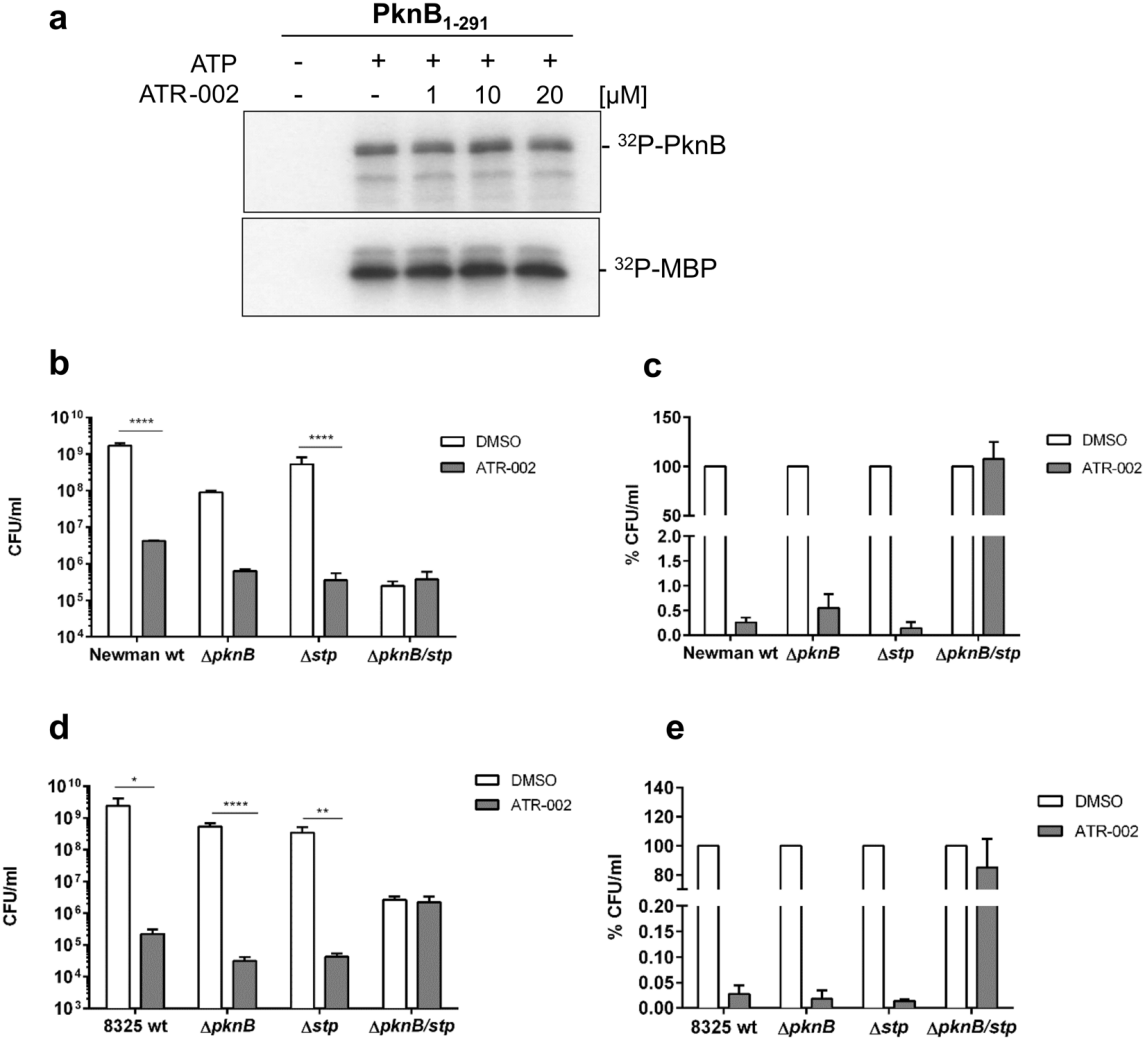
The antibacterial action of ATR-002 is mediated via interaction with signalling pathways of *S*. *aureus*. The impact of ATR-002 on PknB activity was analysed by an *in vitro* kinase assay (**a**). Therefore, 0.5 µg of the purified kinase domain (PknB_1–291_) were incubated in kinase buffer in the presence or absence of 1 µl ATP (2 mM) and 8 µCi ^32^P-ATP and increasing amounts of the MEK-inhibitor ATR-002 (as indicated) for 1 h at 37 °C. The reaction was stopped by addition of 5 × SDS sample buffer. Following SDS page and Western Blot, the phosphorylation of PknB and MBP was analysed by radiography. Additionally, wildtype (WT) or mutant strains lacking either a functional kinase (Δ*pknB*), the phosphatase (Δ*stp*) or both (Δ*pknB/stp)* were treated over-night in the presence of 20 µM ATR-002 or solvent (DMSO) at 37 °C. Afterwards, bacterial titres were calculated by plating serial dilutions on BHI agar plates (**b–e**). Data represent one representative blot of three experiments (**a**) or means + SD of three independent experiments (**b**–**e**). Western Blot data show cropped blots from two full-length blots, which are provided in Figure S8. Statistical significance was analysed by one-way ANOVA followed by Dunnett’s multiple comparisons test (*p < 0.05, **p < 0.01; ***p < 0.001; ****p < 0.0001).

ATR-002 clearly exhibits antibacterial properties, correlating with reduced antibiotic and stress resistance. *S*. *aureus* strains lacking PknB share this phenotype^39–42^. Hence, we addressed whether PknB is involved in the antibacterial action of ATR-002. Therefore, we performed an *in vitro* kinase assay to test for a direct interaction with the purified kinase domain (PknB_1–291_) (generated and provided by the Ohlsen laboratory, Würzburg). The activity of PknB is regulated via autophosphorylation. Thus, we assessed ATR-002 action on PknB autophosphorylation as well as target phosphorylation of MBP (Fig. 5a). In the absence of ATP neither PknB nor MBP were phosphorylated, however, the addition of ATP induced both autophosphorylation of PknB and phosphorylation of MBP. Surprisingly, treatment with ATR-002 did not reduce phosphorylation, indicating that in this system, the inhibitor was not able to block activity of the kinase domain. However, here the influence of the C-terminal domain of PknB or the impact of other bacterial factors that may affect kinase activity during bacterial growth cannot be measured. Therefore, we performed an over-night treatment of different wildtype (WT) *S*. *aureus* strains and respective deletion mutants lacking either the kinase (Δ*pknB*), the corresponding phosphatase (Stp) (Δ*stp*) or both enzymes (Δ*pknB/stp*) with ATR-002 (Fig. 5b–e). ATR-002 treatment clearly reduced bacterial growth of WT strains. Surprisingly, the Δ*pknB* and the Δ*stp* strains were still sensitive to ATR-002. In contrast to that, the double knock-out strains completely lost sensitivity to the inhibitor. These data indicate, that neither PknB nor Stp alone seem to be the targets for the antibacterial action of ATR-002. The inhibitor rather seems to interfere with the complex interplay of both enzymes suggesting that ATR-002 leads to a disturbance of this signalling system.

### The impact of ATR-002 on bacterial growth is not restricted to *S*. *aureus*

**Figure 6:**
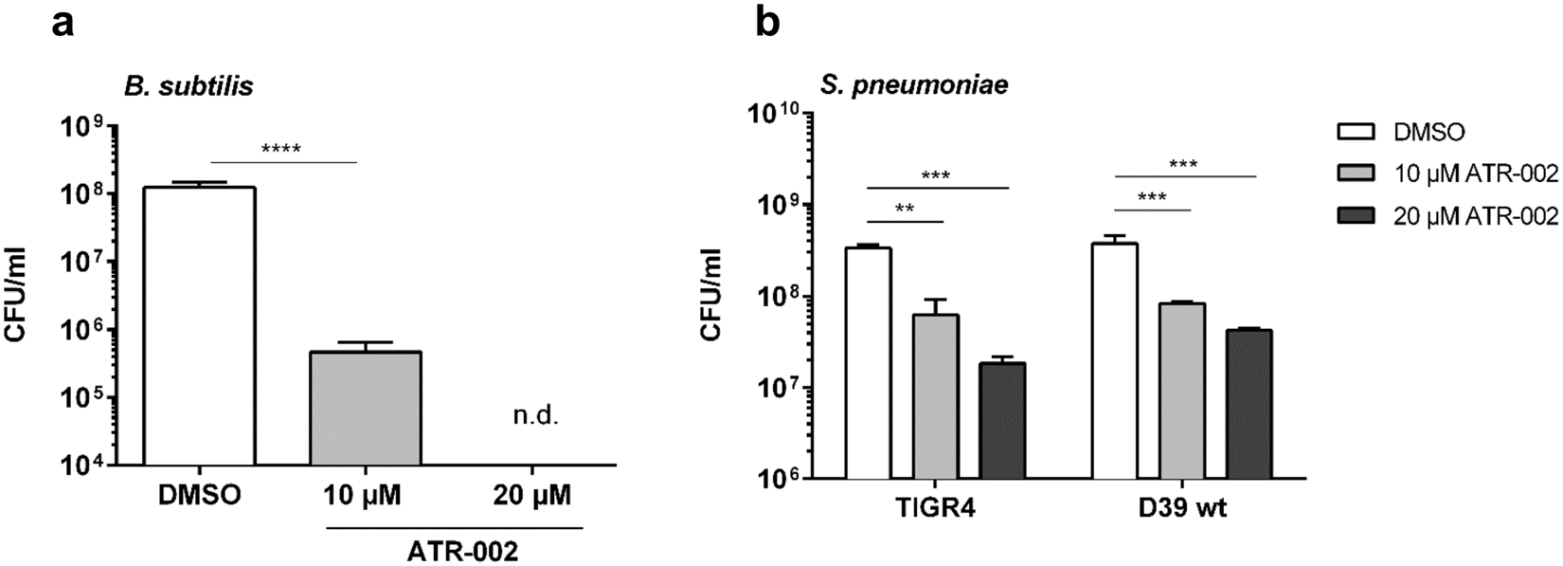
The inhibitory effect of ATR-002 is not restricted to *S*. *aureus*. To test for a potential antibacterial effect on other bacterial species, over-night cultures of *B*. *subtilis* (**a**) and the *S*. *pneumoniae* wild-type strains D39 and TIGR4 (**b**) were incubated with either solvent or different concentrations of the MEK-inhibitor ATR-002 (as indicated) for 18 h. Afterwards, bacterial load was determined via measurement of the OD_600_ and plating of serial dilutions on BHI agar or columbia blood agar plates, respectively. Data represent means + SD of three independent experiments. Statistical significance was analysed by one-way ANOVA followed by Dunnett’s multiple comparisons test (**p < 0.01; ***p < 0.001; ****p < 0.0001).

The presence of bacterial Ser/Thr kinases is described to be conserved among different bacterial genera^25,26,43^. Therefore, we tested whether ATR-002 has antibacterial properties to other bacteria. Hence, the impact on *Escherichia coli* (*E*. *coli*), *Bacillus subtilis* (*B*. *subtilis*) and *Mycobacterium abscessus* (*M*. *abscessus*) was analysed. While growth of *E*. *coli* and *M*. *abscessus* was not affected (not shown), a strong decrease in viable bacterial counts was observed for *B*. *subtilis* in presence of 10 µM ATR-002, and was completely abolished with higher concentrations (Fig. 6a). Besides *S*. *aureus*, one of the most abundant bacteria to cause bacterial super-infections is *S*. *pneumoniae*^44–46^. Thus, activity of ATR-002 against *S*. *pneumoniae* strains D39 and TIGR4 was tested. In the presence of 20 µM ATR-002 a reduction in growth of both strains was observed (Fig. 6b) indicating a broader antibacterial action of ATR-002.

## Discussion

Bacterial super-infections following influenza infections represent an important public health problem and occur during both influenza epidemics and pandemics. Unfortunately, only a limited arsenal of potent anti-infectives against IV and/or *S*. *aureus* is available and drug-resistance is a major concern. For anti-influenza intervention, the use of inhibitors targeting cellular pathways came into the focus of research. Previous studies have shown that blockade of the Raf-MEK-ERK pathway leads to reduced progeny virus production *in vitro* and *in vivo* with a high barrier towards viral resistance^16–18^. However, it remained unclear whether inhibition of this pathway in primary IV infection could predispose the host to subsequent bacterial infections.

While we could not only rule out such a sensitization for bacteria, our findings rather indicate the opposite effect. We show that the metabolic conversion of CI-1040 into its metabolite ATR-002 turns the MEK-inhibitor into an antibacterial compound. This not only resulted in reduced intracellular bacterial titres of *S*. *aureus* in a super-infection scenario but also in strongly reduced bacterial growth in suspension cultures.

The efficacy of cellular MEK-inhibitors against IV infections has been shown in several studies by us and others^16–18^. However, in case of secondary bacterial infections, the impact of MEK-inhibitor treatment has not been analysed so far. We show, that administration of ATR-002 during super-infection of IV and *S*. *aureus* leads to reduced viral titres and inhibition of the CPE. Additionally, intracellular bacterial titres were slightly reduced upon treatment, which could only be observed with ATR-002 and not with CI-1040. Still, these differences in pathogen load do not fully explain the great reduction of the induced CPE. Upon infection, bacteria secrete virulence factors such as toxins, which in turn can lead to tissue damage^36,47,48^. Furthermore, intracellular bacteria can cause cell death upon phagosomal escape^47,49,50^. It cannot be ruled out that administration of ATR-002 might interfere with secretion of bacterial virulence factors and hence, lead to a reduced destruction of surrounding cells and tissue^47,48^. Beside a rather low reduction of intracellular bacterial load, we observed a strong inhibition of bacterial growth in suspension cultures upon ATR-002 treatment correlating with decreased antibiotic and stress resistance. This might be an exclusive function of ATR-002 rather than a general feature of MEK-inhibitors as this strong impairment in growth was not visible with other MEK-inhibitors. The fact that the parental compound CI-1040 did not show any antibacterial effect indicates that metabolic conversion to ATR-002 leads to differences in chemical structure and thereby to higher binding affinity and accessibility to the bacterial target(s). Recently published data show the impact on bacterial growth and pathogenicity in presence of specific inhibitors against the bacterial kinase PknB^41^. Notably, a retention in bacterial growth accompanied by increased antibiotic susceptibility was observed, when bacteria were incubated with a specific Stk1 inhibitor^41^. Furthermore, the dual administration of kinase inhibitors in combination with antibiotics resulted in increased bactericidal properties of ceftriaxone and cefotaxime^41^. Comparable data were also published by Vornhagen *et al*. showing increased antibiotic sensitivity upon inhibitor treatment in case of Methicillin-resistant strains^39^. Our data strongly support the hypothesis that the inhibitory action of ATR-002 is at least partially mediated by interaction with the bacterial PknB-Stp signalling system. It is likely that more than one bacterial target of this system is involved in the observed phenotype. For example, a crosstalk of the PknB-Stp pathway with the two-component system WalKR was demonstrated. This regulator is greatly involved in cell wall synthesis and virulence of *S*. *aureus* and might represent an additional target of ATR-002^51^. Beside the involvement of other pathways, mutations of *S*. *aureus* PknB were shown to affect bacterial cell wall structure^52^. At this point we cannot rule out, if changes in cell wall structure and thickness in bacteria harbouring the double knock-out of PknB and Stp also play a role in the observed phenotype. This might explain the lack of antibacterial activity of ATR-002 in gram-negatives as in these bacteria cell wall structure is completely different to gram-positives like *S*. *aureus*. While the direct target(s) of ATR-002 may not be clear, interference with a more complex system rather than one particular enzyme would have a major advantage: The likeliness of resistance development may be greatly reduced, as it has been observed in our assays. While the antibacterial action of ATR-002 is intriguing, it has to be pointed out that the data provided so far are limited to *in vitro* experiments. The potential of a future therapeutic use needs to be further explored in *in vivo* models, especially in an IV and *S*. *aureus* super-infection model. Another issue that needs final experimental clarification is, whether conversion of CI-1040 to ATR-002 changes its target profile on cellular kinases and pathways. Due to the fact that we do not know the final target of the MEK pathway on viral structures in cells, and crosstalk of the Raf pathway with other signalling cascades has been described^53^, it cannot completely be ruled out that the intracellular anti-pathogen action of ATR-002 involves still other pathways or kinases. Recently we showed that the inhibitor Vemurafenib, that blocks the MEK activator Raf acts antiviral via interference with multiple pathways^54^. While this may serve as an example of crosstalk, it has to be pointed out that Vemurafenib works via a completely different mode of action compared to ATR-002. Furthermore, MEK inhibitors are described to be very specific to their target kinase. Finally, such an intracellular crosstalk would be irrelevant for the direct antibacterial action of ATR-002.

In summary, we show for the first time that metabolic conversion of the cellular MEK-inhibitor CI-1040 into ATR-002 generates an antibacterial compound. Beside interference with the influenza life cycle by interrupting the virus-supportive Raf-MEK-ERK pathway, ATR-002 strongly decreases growth of *S*. *aureus* and other bacterial species. Our findings may have important implications for the development of strategies to treat post-influenza super-infections or infections with antibiotic-resistant bacteria.

## Methods

### Cell lines, viral and bacterial strains

The human alveolar basal lung epithelial cell line (A549) was cultivated in Dulbecco’s Modified Eagle Medium (DMEM) and Madin-Darby canine kidney cells (MDCK) were cultivated in minimal essential medium. Both media were supplemented with 10% fetal bovine serum (FBS).

All viral and bacterial strains used in the present study are listed in Table S4.

Viral stocks were generated by passaging of the viruses on MDCK cells. Infectious particles were calculated via standard plaque assay. Bacteria were stored as glycerol stocks [comprised of brain heart infusion (BHI) and 30% glycerol] at −80 °C and on blood agar plates until further use.

### Super-infection of A549 cells with IV and *S*. *aureus*

An over-night culture of *S*. *aureus* was prepared prior to infection. A single colony was selected and inoculated in 5 ml BHI medium for 16–18 h. Confluent A549 cells were prepared by seeding 6- or 12-well plates (5 × 10^5^ or 2.5 × 10^5^ cells/well) 24 h before the experiment. For viral infection, cells were washed with phosphate-buffered saline (PBS) and infected with 500 µl of the virus stock adjusted in PBS/INF [0.2% bovine serum albumin (BSA), 1 mM MgCl_2_, 0.9 mM CaCl_2_, 100 U/ml penicillin, 0.1 mg/ml streptomycin] to the desired multiplicity of infection (MOI). After 30 min_,_ cells were washed with PBS and the secondary bacterial infection was performed. For this, the indicated MOI was adjusted in 1 ml DMEM/INV [1% human serum albumin, 25 nmol/l HEPES] and cells were incubated for 3 h. To avoid bacterial over-growth, extracellular bacteria were removed by an antibiotic wash step. Therefore, cells were washed with PBS and incubated with 1 ml of DMEM/FBS [10% FBS, 2 μg/ml lysostaphin] for 20 min. Subsequently, cells were washed with PBS and incubated in 1 ml of DMEM/BA [0.2% BSA, 1 mM MgCl_2_, 0.9 mM CaCl_2_] until the end of the experiment. For experiments extending one influenza replication cycle (>8 h) Trypsin solo (2 µg/ml) was added during over-night incubation to allow efficient infection over time. All incubation steps were performed at 37 °C, 5% CO_2._ The infection scheme is summarized in Figure S6.

### Inhibitor treatment during super-infection

The MEK-inhibitors U0126 (Taros Chemicals GmbH & Co. KG), CI-1040 and ATR-002 (originally PD0184264) (kindly supplied by ATRIVA Therapeutics GmbH) were dissolved in DMSO to prepare 10 mM stocks. Before infection, cells were washed with PBS and incubated with 1 ml medium containing the inhibitor (as indicated) or DMSO for 1 h at 37 °C, 5% CO_2_. Afterwards, cells were infected with IV and *S*. *aureus* as described above. During incubation with DMEM/INV and DMEM/BA, media were supplemented with the inhibitors. All inhibitors are described in Table S5. Chemical structures of CI-1040 and the metabolite ATR-002 are displayed in Figure S7.

### Cytotoxicity testing

Potential cytotoxic effects of ATR-002 on A549 cells were determined by counting of viable cells compared to DMSO-treated cells upon 24 and 48 h. Medium containing either DMSO or increasing amounts of ATR-002 was added to A549 cells seeded in 6-well plates and were incubated for 24 or 48 hours. After the respective times viable cells were counted by staining with trypan blue, which selectively stains non-viable cells due to a permissive cell wall. Additionally, supernatants were taken for measurement of LDH release due to membrane rupture. For this, the CytoSelect LDH Cytotoxicity Assay Kit (CBA 241) was used according to manufacturer’s instructions. Values were normalized to DMSO-treated cells and are shown as % viability.

### Calculation of viral and intracellular bacterial titres

For determination of viral replication during the course of infection, the number of infectious particles in the supernatants was measured as described previously^55^. Intracellular bacterial titres were determined by washing infected cells twice with PBS and lysis with 2 ml of ddH_2_O at 37 °C, 5% CO_2_ for 30 min. Afterwards, lysates were transferred into tubes and centrifuged at 4000 rpm, 4 °C for 10 min. Pellets were resuspended in 1 ml PBS, serially diluted and incubated for 24 h at 37 °C on agar plates. Bacterial titres were calculated as colony forming units (CFU)/ml.

### Inhibitor treatment during bacterial growth

U0126, CI-1040 and ATR-002 were added to suspension cultures of *S*. *aureus*, *B*. *subtilis* or *S*. *pneumoniae*. Cultures were prepared by inoculating 5 ml BHI medium with a single bacterial colony and incubating at 37 °C, 5% CO_2_ for 8 h. Subsequently, cultures were centrifuged at 4000 rpm, 4 °C for 5 min and the pellet was washed with PBS. Afterwards, the bacterial suspension was adjusted to an optical density (OD_600_) of 1 in PBS, which represented a viable bacterial count of 5 × 10^8^ CFU/ml in case of *S*. *aureus* (confirmed by plating). For over-night treatment, 5 ml BHI medium were inoculated with 20 CFU/ml and incubated for 16 h in the presence of DMSO, inhibitor (as indicated) or medium at 37 °C, 5% CO_2_. Thereafter, the OD_600_ was measured and cultures were washed with PBS. Bacterial titres were performed by resuspending the pellet in 1 ml PBS and serial dilutions on BHI agar plates. Additionally, growth inhibition was tested in a chemically defined medium (described previously by Schoenfelder *et al*.^56^, RPMI cell culture medium as well as in Mueller-Hinton II medium (Table S5).

### Stress tolerance of *S*. *aureus* after inhibitor treatment

Bacteria were treated over-night in the presence of 20 µM ATR-002 or DMSO as described above. The cultures were then adjusted to OD_600_ = 1 in PBS, diluted 1:100 in BHI medium and incubated for 6 h at 42 °C to induce heat stress. To quantify viable bacteria the cultures were centrifuged at 4000 rpm, 4 °C for 5 min, resuspended in 1 ml PBS, serially diluted and plated on BHI agar plates.

### Time-of-addition assay

For characterization of the anti-bacterial properties of the MEK-inhibitor ATR-002, an over-night culture was prepared. Sub-cultures were prepared by diluting in a ratio of 1:50 in BHI medium. Immediately after inoculation, the cultures were supplemented with DMSO, U0126 (50 µM), ATR-002 (20 µM) or gentamicin (as indicated) and the initial OD_600_ was measured. This measurement was repeated every three hours until 9 h post inoculation. Each culture was then diluted in BHI medium (1:500) and incubated over-night at 37 °C, 5% CO_2_. Finally, the OD_600_ of each culture was measured again.

### Antimicrobial susceptibility testing

Bacterial cultures were treated over-night with 20 µM ATR-002, DMSO or medium as described above. The cultures were then centrifuged at 4000 rpm, 4 °C for 5 min and the bacterial pellet resuspended in 1 ml PBS. Antimicrobial susceptibility testing was then prepared with 100 µl aliquots of each culture on BHI agar plates, followed by application of M.I.C.Evaluator strips (purchased from Oxoid) onto the agar. Plates were incubated at 37 °C for 24 h. The concentration preventing bacterial growth was termed as the minimal inhibitory concentration (MIC) for each individual antibiotic tested.

### *In vitro* kinase assay

For performing an *in vitro* kinase assay, 0.5 µg of the purified bacterial kinase domain (PknB_1–291_) were incubated in 30 µl kinase buffer [50 mM Tris/HCl (pH 7.5), 3 mM MgCl_2_, 3 mM MnCl_2_ and 1 mM DTT] +/− 1 µl ATP (2 mM), +/− 8 µCi ^32^P-ATP together with 4 µg of the substrate MBP for 1 h at 37 °C in the presence of different concentrations of ATR-002 (as indicated) or kinase buffer alone. The reaction was stopped by addition of 5 × SDS sample buffer, denaturation of the proteins at 95 °C for 10 min. Thereafter, proteins were analyzed by SDS-PAGE and Western Blot. Phosphorylated protein bands were visualized by radiography.

### Statistical analysis

All experiments were performed at least three times. Results are presented as mean + SD. Statistical significance was calculated with GraphPad Prism software versions 6 using the indicated statistical tests. *P*-values are indicated by asterisks *p < 0.05; **p < 0.01; ***p < 0.001; ****p < 0.0001.

### Data availability

The datasets generated during the current study are available from the corresponding author on reasonable request.

## Electronic supplementary material

Supplementary information
