# Supplementary material for: Metabolic conversion of CI-1040 turns a cellular MEK-inhibitor into an antibacterial compound
Source: Sci Rep. 2018 Jun 14;8:9114. doi: 10.1038/s41598-018-27445-7 (PMC6002397; doi:10.1038/s41598-018-27445-7)
Supplement: Supplementary file 1 — Supplementary information [file 41598_2018_27445_MOESM1_ESM.pdf]

Metabolic conversion of CI-1040 turns a cellular MEK-inhibitor into an antibacterial compound.

Christin Bruchhagen[1], Marcel Jarick[2], Carolin Mewis[1], Tobias Hertlein[2], Silke Niemann[3], Knut Ohlsen[2], Georg Peters[3],Oliver Planz[4], Stephan Ludwig[1] and Christina Ehrhardt[1,*]

Supplementary Information

Supplementary figures

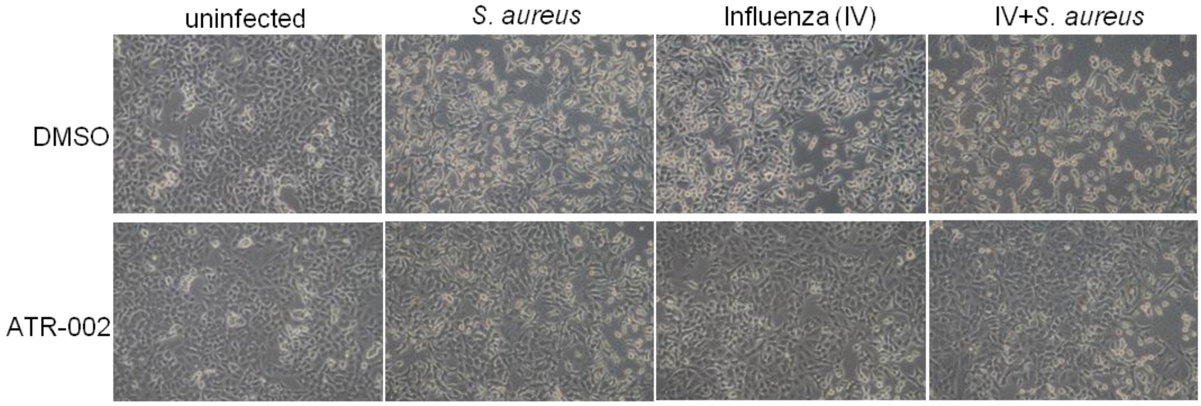

Figure S1: Treatment with ATR-002 strongly decreases the pathogen-induced CPE upon singular bacterial/viral and co-infection. A549 cells were pre-treated for 1 h with 10 µM of the specific MEK-inhibitor ATR-002 or DMSO as solvent control. Afterwards, cells were rinsed with PBS and infected with influenza virus (IV) (MOI as indicated) for 30 min at 37 °C, 5% CO_2. Subsequently, cells were washed with PBS and infected with *S. aureus* 6850 (MOI as indicated) in the presence or absence of the inhibitor for 3 h. To avoid bacterial over-growth, an antibiotic wash step with lysostaphin (2 µg/mL) was performed for 20 min at 37 °C to remove not-internalized bacteria. Then, cells were washed once and were further incubated until 24 h p.i. in the presence of the inhibitor or solvent. At the end of the incubation period the cell monolayer was analyzed via light microscopy.

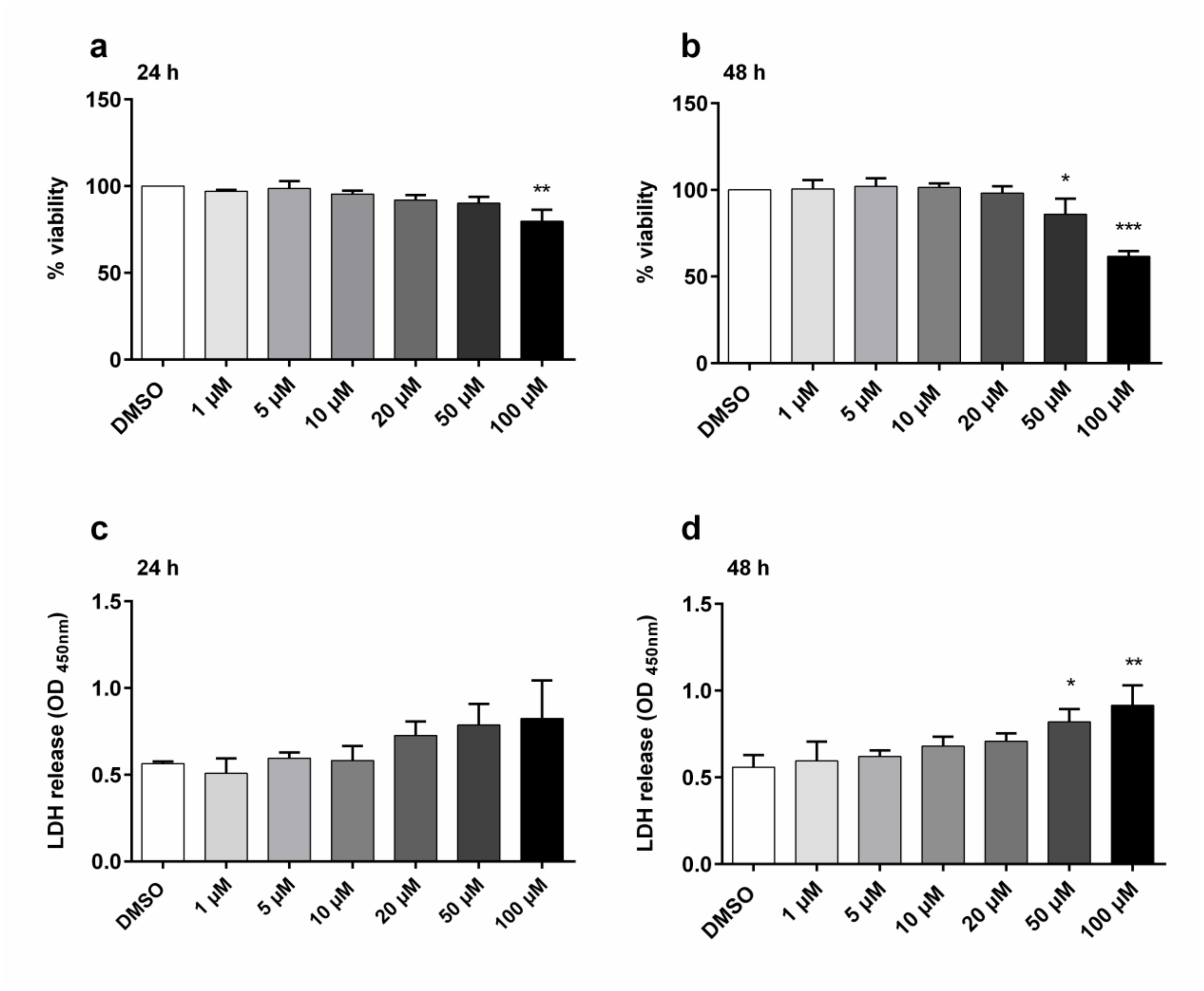

Figure S2: Treatment of A549 cells with ATR-002 does not induce cell toxicity.

A549 cells were treated for 24 (a, c) or 48 (b, d) hours with increasing concentrations of ATR-002 (as indicated). After the incubation times, supernatants were taken for measurement of LDH release (c, d) using the CytoSelect LDH Cytotoxicity Assay Kit according to the manufacturer`s instructions. Additionally, viable cells were counted by staining with trypan blue. Cell viability was normalized to DMSO-treated cells and is shown as % viability. Data show means + SD of three independent experiments. Statistical significance was calculated by one-way ANOVA followed by Dunnett`s multiple comparisons test (* $p < 0.05$; ** $p < 0.01$; *** $p < 0.001$).

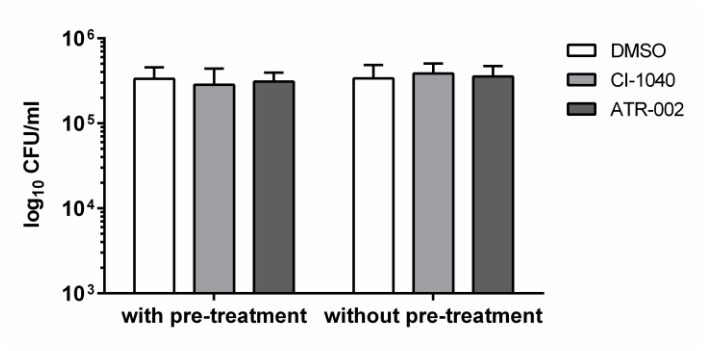

Figure S3: Neither pre-treatment of A549 cells nor administration of ATR-002 or CI-1040 during infection affects bacterial infectivity.

A549 cells were either pre-treated with DMSO, 10 µM CI-1040, 10 µM ATR-002 or were left untreated for 1 h. Then, cells were infected with *S. aureus* 6850 for 2 h (MOI 10) at 37 °C. Afterwards, extracellular bacteria were removed by an antibiotic wash step with lysostaphin for 20 min. Cells were lysed and viable intracellular bacterial titres were determined by plating of serial dilutions. Data represent means + SD of two independent experiments.

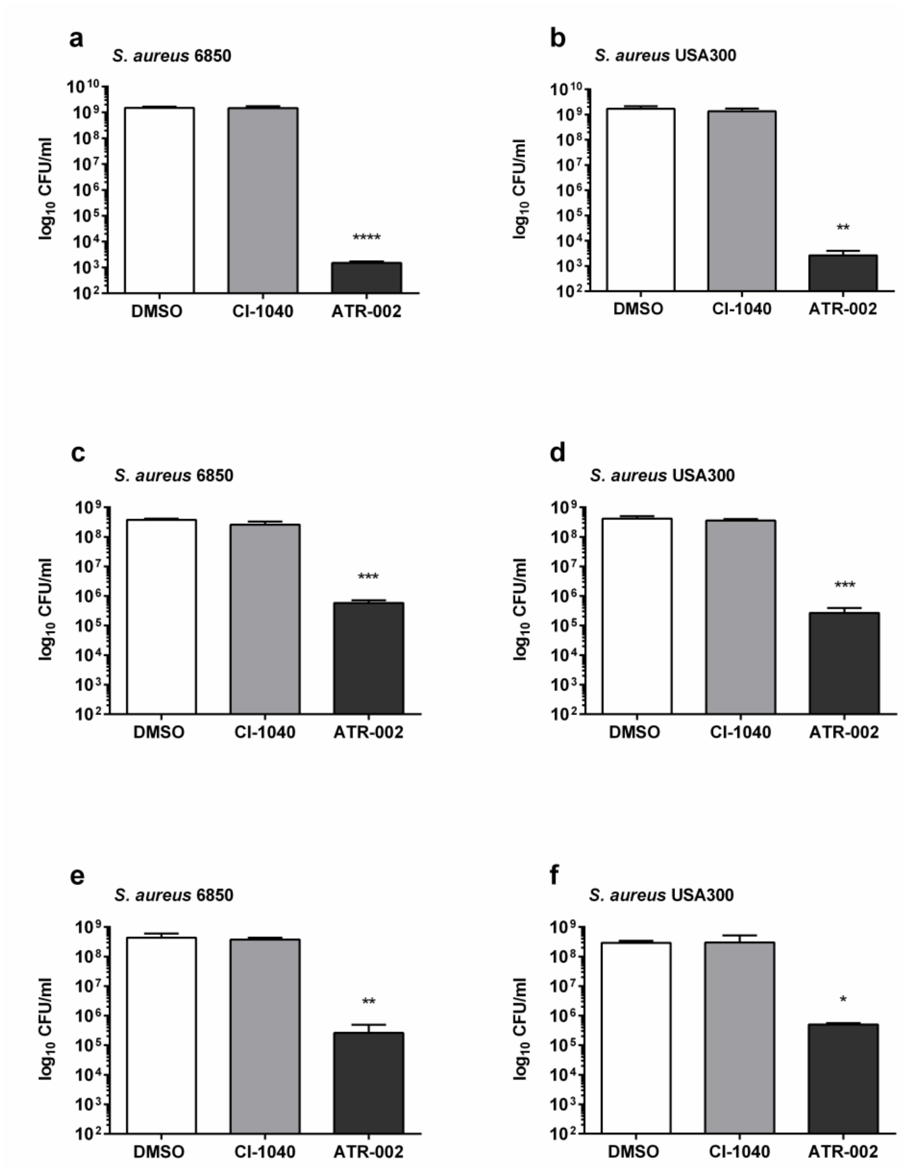

Figure S4: The antibacterial action of ATR-002 is medium-independent.

Over-day cultures of *S. aureus* 6850 or the MRSA strain USA300 were set to 20 CFU/ml and treated with either DMSO, 20 µM of CI-1040 or ATR-002 diluted in a chemically defined minimal medium (a, b), RPMI cell culture medium (c, d) or Mueller-Hinton II medium over-night at 37 °C and 5% CO_2. Serial dilutions were subjected to agar plates to calculate viable bacterial titres shown as colony forming units per ml (CFU/ml). Data represent means + SD of three independent experiments. Statistical significance was evaluated by one-way ANOVA followed by Tukey`s multiple comparisons test (* $p < 0.05$; ** $p < 0.01$; *** $p < 0.001$; **** $p < 0.0001$).

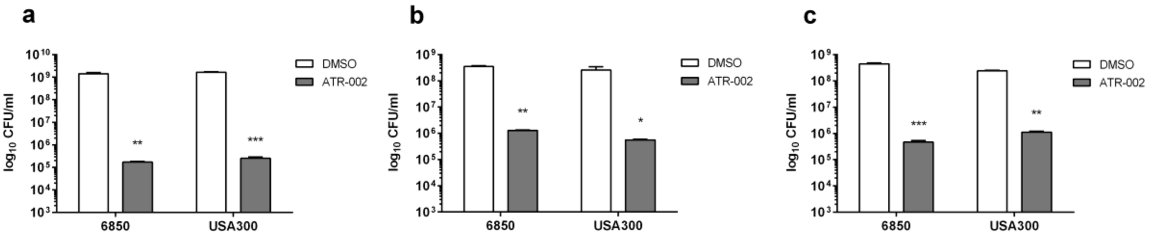

Figure S5: Changes in heat stress response induced by ATR-002 are independent of the bacterial growth medium.

Solvent (DMSO) or inhibitor (20 µM ATR-002) treated over-night cultures of *S. aureus* MSSA (6850) or MRSA (USA300) strains were further cultivated after dilution in a chemically defined minimal medium (a), RPMI cell culture medium (b) or Mueller-Hinton II medium (c) at 42 °C to induce heat stress for 6 h. Then, bacterial counts were analyzed. Data represent means + SD of three individual experiments. Statistical significance was analyzed by one-way ANOVA followed by Dunnett`s multiple comparisons test (** $p < 0.01$; *** $p < 0.001$).

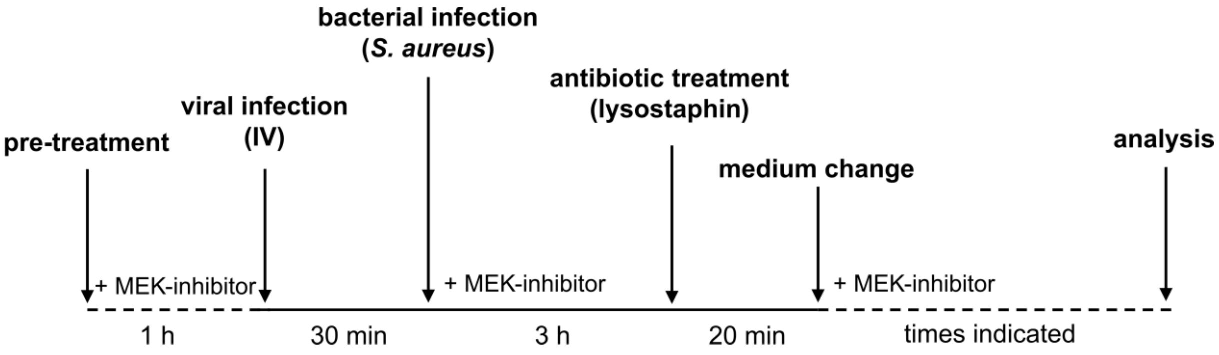

Figure S6: Scheme of the super-infection protocol in A549 cells.

A549 cells were pre-treated for 1 h with 10 µM of the specific MEK-inhibitors CI-1040, ATR-002 or DMSO as solvent control. Afterwards, cells were rinsed with PBS and infected with influenza virus (IV) (MOI as indicated) for 30 min at 37 °C, 5% CO_2. Subsequently, cells were washed with PBS and infected with *S. aureus* 6850 (MOI as indicated) in the presence or absence of the inhibitors for 3 h. To avoid bacterial over-growth, an antibiotic wash step with lysostaphin (2 µg/mL) was performed for 20 min at 37 °C to remove non-internalized bacteria. Then, cells were washed once and were further incubated until 24 h p.i. in the presence of the inhibitors or solvent and were used for further analysis.

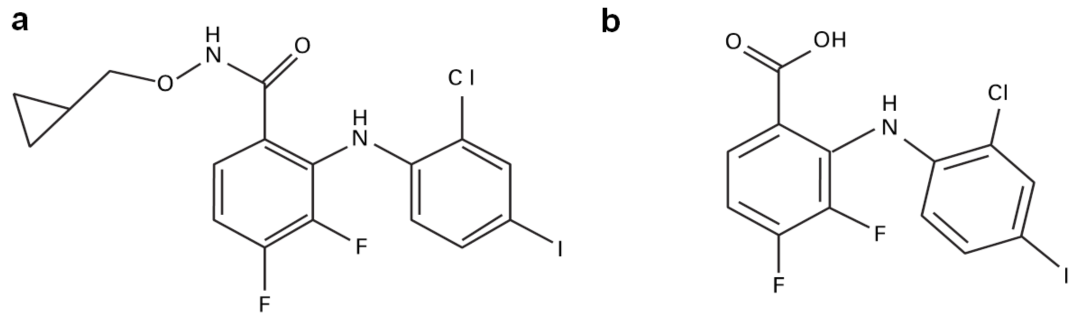

Figure S7: Structural formula of the MEK-inhibitors CI-1040 (a) and its active acidic metabolite ATR-002 (PD0184264) (b).

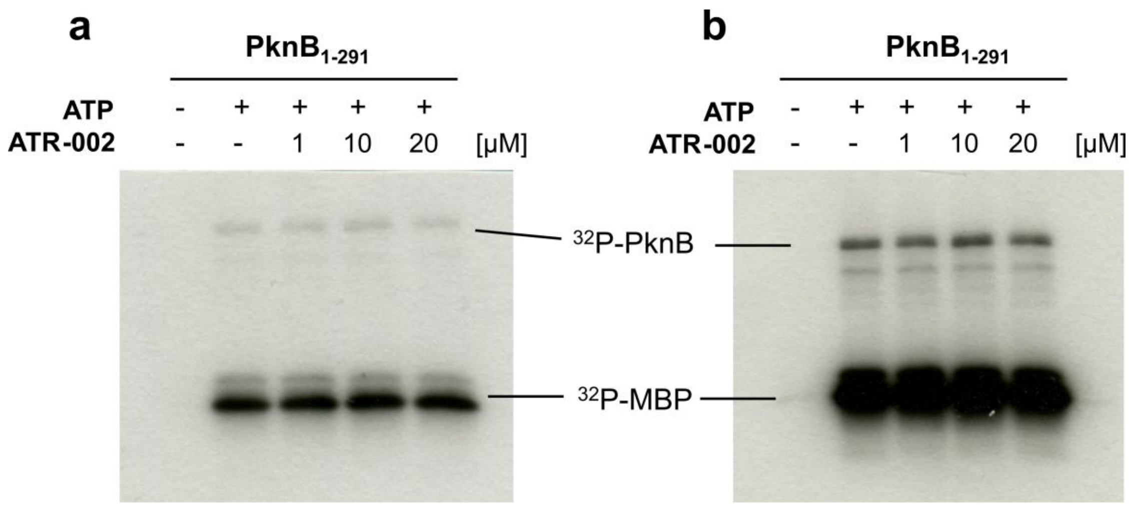

Figure S8: Kinase assay in presence or absence of ATR-002. The impact of ATR-002 on PknB activity was analyzed by an *in vitro* kinase assay. Therefore, 0.5 µg of the purified kinase domain ($PknB_{1\text{-}291}$) were incubated in kinase buffer in the presence or absence of 1 µl ATP (2 mM) and 8 µCi ^{32}P-ATP and increasing amounts of the MEK-inhibitor ATR-002 (as indicated) for 1 h at 37 °C. The reaction was stopped by addition of 5 × SDS sample buffer. Following SDS page and Western Blot, the phosphorylation of PknB and MBP was analyzed by radiography. Original blots are shown after an exposure time of 2 h (a) and 14 h (b).

Supplementary tables

Table S1 Average MICs after analysis of resistance development (cross-resistance)

	Minimal inhibitory concentration (MIC) [mg/L]			
	untreated	Gentamicin-treated	Erythromycin-treated	ATR-002-treated
Gentamicin	2	8	2	0.25
Penicillin	16	16	16	0.5
Meropenem	0.06	0.06	0.06	0.03
Linezolid	2	2	2	1
Ciprofloxacin	0.25	0.25	0.25	0.25

***** results represent average values of three individual experiments

Table S2 Determination of MICs after over-night treatment with ATR-002

	Minimal inhibitory concentration (MIC) [mg/L]			
	S. aureus 6850		*S. aureus* USA300	
	untreated	ATR-002	untreated	ATR-002
Penicillin	16	0.25 - 0.5	> 256	8
Meropenem	0.06	0.03	0.12 - 0.25	0.06
Linezolid	2	1	2	1
Ciprofloxacin	0.25	0.12 - 0.25	8	4 - 8
Gentamicin	2	0.12	1	0.12

***** results represent average values of three individual experiments

Table S3 Determination of MICs for representative antibiotics after over-night treatment with ATR-002 in Mueller-Hinton II medium

	Minimal inhibitory concentration (MIC) [mg/L]			
	S. aureus 6850		*S. aureus* USA300	
	untreated	ATR-002	untreated	ATR-002
Penicillin	4	0.12	> 256	8
Linezolid	2	0.5	2	1
Gentamicin	1	0.12	1	0.12

***** results represent average values of two individual experiments

Table S4 Summary of viral and bacterial strains used within the present study

Viral/bacterial strain	Classification	origin
H7N7	Influenza A/Seal/Massachusetts/1/80 (H7N7, SC35M)	Institute of Virology Muenster
H1N1	Influenza A/Puerto Rico/8/34	Institute of Virology

	(H1N1, PR8M - Münster)	Muenster
S. aureus 6850	MSSA; wildtype	Institute of Medical Microbiology Muenster
S. aureus USA300	MRSA; wildtype	Institute of Medical Microbiology Muenster
S. aureus 8325	MSSA; wildtype	Institute of Molecular Infection Biology Wuerzburg
S. aureus 8325 *ΔpknB*	MSSA; deletion of Ser/Thr kinase PknB	Institute of Molecular Infection Biology Wuerzburg
S. aureus 8325 *Δstp*	MSSA; deletion of phosphatase Stp	Institute of Molecular Infection Biology Wuerzburg
S. aureus 8325 *ΔpknB/stp*	MSSA; deletion of Ser/Thr kinase PknB and phosphatase Stp	Institute of Molecular Infection Biology Wuerzburg
S. aureus Newman HG	MSSA; wildtype	Institute of Molecular Infection Biology Wuerzburg
S. aureus Newman HG *ΔpknB*	MSSA; deletion of Ser/Thr kinase PknB	Institute of Molecular Infection Biology Wuerzburg
S. aureus Newman HG *Δstp*	MSSA; deletion of phosphatase Stp	Institute of Molecular Infection Biology Wuerzburg
S. aureus Newman HG *ΔpknB/stp*	MSSA; deletion of Ser/Thr kinase PknB and phosphatase Stp	Institute of Molecular Infection Biology Wuerzburg
Bacillus subtilis	/	Institute of Medical Microbiology Muenster
Mycobacterium abscessus	/	Institute of Medical Microbiology Muenster
Escherichia coli	/	Institute of Medical Microbiology Muenster
Streptococcus pneumoniae D39	serotype 2; wildtype	Prof. Dr. Sven Hammerschmidt, Greifswald
Streptococcus pneumoniae TIGR4	serotype 4; wildtype	Prof. Dr. Sven Hammerschmidt, Greifswald

Bacterial species names are shown in italic style.
MSSA - Methicillin-sensitive *Staphylococcus aureus*
MRSA - Methicillin-resistant *Staphylococcus aureus*

Table S5 Summary of media and inhibitors used in the present study

Substance	classification/origin
BHI medium	Merck
THY medium	THB medium (Sigma-Aldrich) + 2% yeast extract (BD)
Columbia blood agar	BD
Chemically defined minimal medium	prepared and provided by the Institute of Molecular Infection Biology Wuerzburg according to Schoenfelder et al. 2013
Mueller-Hinton II medium/agar	BD

BHI agar plates	BHI Medium (Merck) + 17 g Bacto Agar (BD)
RPMI cell culture medium	Sigma Aldrich (R8758-500ML)
U0126 (MEK-inhibitor)	1,4-Diamino-2,3-dicyano-1,4-*bis* (2-aminophenylthio)butadiene (Taros Chemicals GmbH & Co. KG)
CI-1040 (MEK-inhibitor)	2-(2-Chloro-4-iodophenylamino)-N-cyclopropylmethoxy-3,4-difluorobenzamide (ATRIVA Therapeutics GmbH)
ATR-002 (originally termed as PD0184264) (MEK-inhibitor, active metabolite of CI-1040)	2-[(2-Chloro-4-iodophenyl)amino]-3,4-difluoro-benzoic acid (ATRIVA Therapeutics GmbH)
Gentamicin	Applichem
Erythromycin	Sigma-Aldrich
Lysostaphin	Sigma-Aldrich
M.I.C.Evaluator (M.I.C.E.) strips	Oxoid
CytoSelect LDH Cytotoxicity Assay Kit	Cell Biolabs inc. (CBA-241)

98
